# Skull Base Repair following Resection of Vestibular Schwannoma: A Systematic Review (Part 1: The Retrosigmoid Approach)

**DOI:** 10.1055/a-2222-0184

**Published:** 2024-01-22

**Authors:** Joachim Starup-Hansen, Simon C. Williams, Alexandra Valetopoulou, Danyal Z. Khan, Hugo Layard Horsfall, Jigishaa Moudgil-Joshi, Oliver Burton, Hala Kanona, Shakeel R. Saeed, William Muirhead, Hani J. Marcus, Patrick Grover

**Affiliations:** 1 Victor Horsley Department of Neurosurgery, The National Hospital for Neurology and Neurosurgery, University College London NHS Trust, London, United Kingdom; 2 Wellcome/EPSRC Centre for Interventional and Surgical Sciences, University College London, London, United Kingdom; 3 Department of Neurosurgery, The Royal London Hospital, London United Kingdom; 4 Department of Neurosurgery, Charing Cross Hospital, London, United Kingdom; 5 The Royal National Throat, Nose and Ear Hospital, London, United Kingdom; 6 University College London Ear Institute, London, United Kingdom

**Keywords:** vestibular schwannoma, CSF leak, skull base repair, neurosurgery, ear nose and throat, retrosigmoid approach

## Abstract

**Objective** Despite advances in skull-base reconstruction techniques, cerebrospinal fluid (CSF) leaks remain a common complication following retrosigmoid (RS) vestibular schwannoma (VS) surgery. We aimed to review and classify the available strategies used to prevent CSF leaks following RS VS surgery.

**Methods** A systematic review, including studies of adults undergoing RS VS surgery since 2000, was conducted. Repair protocols were synthesized into a narrative summary, and a taxonomic classification of techniques and materials was produced. Additionally, the advantages, disadvantages, and associated CSF leak rates of different repair protocols were described.

**Results** All 42 studies were case series, of which 34 were retrospective, and eight were prospective. Repair strategies included heterogeneous combinations of autografts, xenografts, and synthetic materials. A repair taxonomy was produced considering seven distinct stages to CSF leak prevention, including intraoperative approaches to the dura, internal auditory canal (IAC), air cells, RS bony defect, extracranial soft tissue, postoperative dressings, and CSF diversion. Notably, there was significant heterogeneity among institutions, particularly in the dural and IAC stages. The median postoperative incidence of CSF leaks was 6.3% (IQR: 1.3–8.44%).

**Conclusions** The intraoperative strategies used to prevent CSF leaks during RS VS surgery vary between and within institutions. As a result of this heterogeneity and inconsistent reporting of CSF leak predictive factors, a meaningful comparative analysis of repair protocols was not feasible. Instead, we propose the development of a prospective multicenter observational evaluation designed to accurately capture a comprehensive dataset of potential CSF risk factors, including all stages of the operative repair protocol.

## Introduction

The retrosigmoid (RS) approach is a versatile surgical corridor used to treat a variety of lesions of the cerebellopontine angle, including vestibular schwannomas (VSs). Since the first description of VS in the latter half of the 18th century, advances in operative resection techniques have contributed to dramatic improvements in mortality rates. 1 2 However, surgical resection requires an iatrogenic disruption of the lateral skull base, introducing the risk of complications such as cerebrospinal fluid (CSF) leaks. CSF leaks represent the most prevalent postoperative complication following RS VS surgery, affecting ∼10% of cases and contributing to life-threatening conditions such as meningitis, wound infections, prolonged hospitalizations, recurrent surgical interventions, and a consequential increase in healthcare expenditures. 3 4 5 6

Numerous factors influence the incidence of postoperative CSF leak, including patient factors, choice of approach, and the method of skull base repair. 7 8 While certain factors are non-modifiable, the operative repair protocol remains within the surgeon's control. Indeed, several refinements of the surgical closure technique have been introduced in recent decades, often catalyzed by the development of biomaterials to be used in the various stages of reconstruction. Such reconstructive materials and techniques vary, and include the solitary or combined use of autografts, xenografts, and synthetic substitutes. Additionally, pressure reducing strategies via CSF diversion (i.e., lumbar drains) may also be used. 9 As a result of the many strategies available, the optimal combination of techniques and materials remains unclear.

To determine the optimal protocol for preventing CSF leaks, the present systematic review offers a comprehensive classification of skull base repair strategies following VS resections performed via the RS approach. Our goal is to elucidate the advantages, disadvantages, and outcomes associated with each repair technique, guiding surgeons in making informed decisions and shaping future prospective evaluations.

## Methods

A PRISMA adherent systematic review of the literature was performed. This publication is part 1 of a two-part series considering skull base repair techniques for VS surgery via the RS and translabyrinthine approaches, respectively. A study protocol was generated prior to data collection (PROSPERO ID: CRD42023388777).

### Search Strategy

The search strategy encompassed synonymous terms for “VS,” “retrosigmoid,” and “CSF leaks”; a detailed search strategy can be found in Supplementary Table S1 (available in the online version only). Studies were included if they (1) were published in English from 2000 to 2023, (2) reported a technique for skull base repair following the resection of VS via the RS approach, and (3) included the incidence of postoperative CSF leakage of any kind, including otorhinorrhea and external CSF leaks. Exclusion criteria were case series with fewer than three VS patients, conference abstracts, editorials, reviews, animal studies, and cadaveric studies. Studies reporting multiple surgical approaches (e.g., translabyrinthine approach, middle fossa approach) were included only if they reported CSF-related outcomes for each approach separately; papers that provided combined leak rates of different surgical corridors were excluded. Studies reporting non-VS indications for RS surgery were included as long as VS made up at least three cases (consistent with our case series limit). PubMed and EMBASE databases were searched on March 15, 2023. Citation references of included studies were reviewed for additional candidate articles.

OVID and Rayyan (version 9.4.1) were used for de-duplication. Abstract screening was conducted by two independent reviewers in duplicate (J.S.-H., S.C.W.). Any conflicts between reviewers were resolved through arbitration by a third author (H.J.M.).

### Data Extraction

Extracted data points of included studies consisted of study details (design, follow-up length), patient demographics (e.g., sample size, age, sex), tumor characteristics (size), CSF preventative strategies (techniques, materials), strategy rationales, CSF leak diagnostic criteria, CSF leak rates, and the treatment strategies following confirmation of CSF leaks. If studies reported multiple techniques with individual cohort descriptions, this was reflected in the data extraction.

### Quality Assessment

Risk of bias was analyzed using a bespoke tool adapted from a prior systematic review of endonasal skull base reconstructive strategies conducted by our group. 10 The tool is based on COSMOS-E guidance and interrogates key study properties, including the clarity of reporting of CSF leak risk factors, treatment groups, repair strategies, and outcome definitions. 11 Studies were rated out of 5 and stratified according to lowest risk (score 0–1) and highest risk (scores 4–5).

### Data Analysis

Data was analyzed using Excel (Microsoft, version 16.66) and combined into a narrative synthesis, outlining the techniques and materials used to prevent CSF leaks following RS VS surgery. Such a synthesis was used to produce a taxonomic classification of repair strategies, with subgroupings based on the anatomic level of repair. Additionally, the frequency of techniques was described. However, no attempt was made to comment on the superiority of the various strategies—except in circumstances where individual studies identified drawbacks or benefits of a technique. The incidence of CSF leaks was analyzed using descriptive statistics (median, interquartile range) to account for the heterogenous inclusion criteria and possible overlap of patient cohorts by some groups, limiting the validity of a pooled synthesis.

## Results

### Overview

**Fig. 1:**
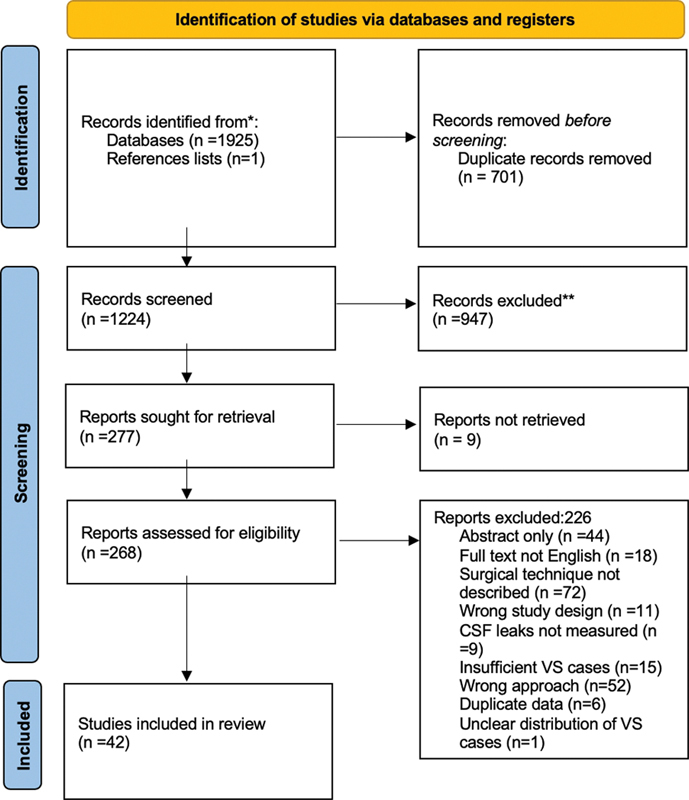
PRISMA flow diagram describing the progressive exclusion of candidate articles from the title screen to the full-text review.

The search identified 1,925 articles, of which 42 were included for full-text analysis ( Fig. 1 ). Eight studies had at least one arm that was prospective, 12 13 14 15 16 17 34 studies were retrospective. Eight out of 42 studies compared different techniques or materials. 9 15 17 18 19 20 21 22 23 Fifteen out of 42 studies included non-VS indications for the RS approach, which are listed in Table 1 . 5 7 14 19 21 24 25 26 27 28 29 30 31 32 33 The annual rate of publication increased over time; the first 5 years of analysis (2000–2004) returned four publications meeting the inclusion criteria, whereas the last 5 years (2019–2023) returned 13. The median risk of bias was 2/5 (IQR: 1–3), suggesting moderate risk of bias ( Supplementary Table S2 [available in the online version only]). Most studies were from groups out of North America (18/42, 43%), followed by Europe (14/42, 33%), Asia (9/42, 21%), and Africa (1/42, 2%).

**Table 1:** Study demographics, ordered by study design

Authors	Year	Study type	Subtypes of repair technique (if multiple arms)	Total patients	Age in years (mean)	Sex (M:F)	Pathologies	Mean tumor size (mm)	BMI	Follow-up (mean)
Jung et al 47	2000	Retrospective case series		30	45.2	12 M:18 F	VS	49.2	NS	1 y +
Leonetti et al 28	2001	Retrospective case series		*191*	56.4 a	1 M:1.18 F a	77% VS a	NS	NS	NS
Bani and Gilsbach 41	2002	Retrospective case series		224	NS	NS	VS	NS	NS	NS
Becker et al 50	2003	Retrospective case series		100	48	52 M:48 F	VS	24	NS	NS
Fishman et al 37	2004	Retrospective case series		71	NS	NS	VS	NS	NS	
Cueva and Mastrodimos 48	2005	Retrospective case series		115	NS	NS	VS	**NS**	NS	NS
Samii et al 43	2006	Retrospective case series		200	46.8	NS	VS	NS	NS	24 mo
Lüdemann et al 23	2008	Retrospective case series	*Air cells: fat plug*	137	NS	73 M:64 F	VS	NS	NS	NS
			*Air cells: muscle plug*	283	NS	140 M 143 F	VS	NS	NS	NS
Bayazit et al 26	2009	Retrospective case series		412	42.9	194 M:218 F	VS = 137, Meniere's = 234, recurrent vestibulopathy = 3, meningioma = 3, arachnoid cyst = 1	NS	NS	NS
Della Pepa et al 19	2011	Retrospective case series	*Craniectomy*	20	53.6	18 M:16 F	VS = 11, nerve decompression = 15; hemifacial spasm = 3; epidermoid cyst = 3; arachnoid cyst = 1; glossopharyngeal neuralgia = 1	NS	NS	5.02 y
			*Craniotomy*	14						
Stieglitz et al 40	2011	Retrospective case series		519	49	263 M:256 F	VS	NS	NS	NS
Arlt et al 20	2011	Retrospective case series	*Sandwich dural closure*	41	59.7	18 M:23 F	VS	25 b	NS	26.4 mo b
		Retrospective case series	Onlay dural closure	40	55	16 M:24 F	VS	22.5 b	NS	
Ling et al 24	2014	Retrospective case series		58	52.1	20 M:38 F	Trig. neuralgia = 22, VS = 18, p fossa meningioma = 12, epidermoid tumor = 4, arachnoid cyst = 1, cerebellar abscess = 1, skull base dermoid tumor = 1, hemangioblastoma = 1, cerebral cyst = 1, small cell met = 1	NS	NS	12.4 mo
Daming et al 39	2014	Retrospective case series		37	45.1	16 M:21 F	VS	47	NS	NS
Crowson et al 9	2016	Retrospective case series	*Preoperative LD*	82	52.3 a	1 M:1.33 F a	VS	19.1 a	NS	NS
			*No LD*	48	52.3 a	1 M: 1.33 F a	VS	19.1 a	NS	NS
Azad et al 34	2016	Retrospective case series		24	47	10 M:14 F	VS	Stage 1 tumor (4.2%), Stage 2 (33.3%), Stage 3 (25%), Stage 4 tumors (37.5%)	NS	20.6 mo
Mastronardi et al 33	2016	Retrospective case series		27	20–77 (range)	12 M:15 F	12 VS, 4 CPA meningiomas 9 MVD, 1 hemifacial spasm, 1 PICA aneurysm	NS	NS	NS
Luryi et al 27	2017	Retrospective case series		20	48.9	6 M:4 F	VS = 5, MVD = 9, epidermoid cyst = 2, meningioma = 2, IAC osteoma = 2	NS	NS	9.8 mo b
Goodarzi et al 31	2018	Retrospective Case Series		25	NS	NS	CP angle schwannoma = 8, hemifacial spasm = 4, CP angle meningioma = 3, epidermoid cyst = 3, Meniere's = 3, arachnoid cyst = 1, CP angle calcifying pseudoneoplasm = 1, petroclival meningioma = 1, trigeminal neuralgia = 1	NS	NS	NS
Venable et al 32	2018	Retrospective case series		86	55 b	33 M:53 F	MVD = 50, VS = 10, mets = 7, meningioma = 7, glioma = 3, chordoma = 1, dermoid cyst = 2, brainstem cavernous malformation = 2, cerebellar abscess = 1	NS	NS	NS
Chen et al 30	2019	Retrospective case series		97	51.8	42 M:55 F	VS ( *n* = 49), meningioma ( *n* = 19), epidermoid cyst ( *n* = 10), cavernous hemangioma ( *n* = 5), glioma ( *n* = 14)	NS	NS	NS
Ou et al 21	2019	Retrospective case series	*Autologous bone flap*	*107*	58 b	NS	VS = 6, TN = 41; HFS = 58; masticatory muscle spasm = 1; intermediate nerve neuralgia = 1	NS	NS	13 mo
			*PPMA cement*	*136*	58.5 b	NS	VS = 3, TN = 58; HFS = 71; epidermoid cyst = 4	NS	NS	12 mo
Sathaporntheera and Saetia 5	2020	Retrospective case series		286	51.8	92 M:194 F	VS = 152, TN = 42, hemifacial spasm = 28, meningioma = 52, other = 12	NS	24.15	NS
Plainfossé et al 45	2022	Retrospective case series		175	53.6	4.55 M:1 F a	VS	NS	25.7	5.5 y
Montano et al 7	2021	Retrospective case series		103	53.6	47 M:56 F	VS = 62, meningioma = 7, trig neuralgia = 24, schwannoma CNV = 3, schwannoma other = 7	NS	NS	35.6 mo
Schackert et al 49	2021	Retrospective case series		544	57 b	245 M:299 F	VS	The majority (77%) T3 and T4 tumors	NS	6 mo
Zhang et al 46	2021	Retrospective case series		177	52.4	82 M:95 F	VS	22	NS	Minimum 1 y
Hwa et al 18	2021	Retrospective case series	*No bone cement*	32	45	20 M:12 F	VS	25	28.9	NS
			*Bone cement (Norian)*	63	49	21 M:42 F	VS	22.6	27.2	NS
			*Bone cement (Cranios)*	101	53	39 M:62 F	VS	22.3	28.8	NS
Magill et al 29	2021	Retrospective case series		40	54 b	16 M:34 F	VS = 38%, meningioma = 43%, IgG4-related disease = 5%, epidermoid cyst = 3%, neuroenteric cyst = 3%, craniopharyngioma = 3%, metastatic carcinoma = 3%, hemangioblastoma = 3%, choroid plexus papilloma = 3%	NS	NS	NS
Yang et al 51	2023	Retrospective case series		16	33–75	9 M:7 F	VS	43.6	NS	Range: 3–24 mo
Wong and Wong 25	2023	Retrospective case series		114	60.8	40 M:74 F	Trig. neuralgia = 49, hemifacial spasm = 25, Meniere's = 2, vestibular schwannoma = 6, meningioma = 14, epidermoid = 2, other = 16	NS	28.4	54 wk
Chibbaro et al 15	2018	Prospective case series	*Lazy* ***S*** *-shaped incision*	40	40	26 M:14 F	VS	NS	NS	NS
			*Modified* ***C*** *-shaped incision*	40	43	22 M:18 F	VS	NS	NS	NS
Brennan et al 35	2001	Prospective case series		151	NS	NS	VS	8.5	NS	NS
Kalamarides et al 16	2004	Prospective case series		59	NS	NS	VS	NS	NS	1 y +
Baird et al 17	2007	Prospective case series	*HAC group*	130	49	NS	VS	21		3.8 mo b
			*Traditional IAC treatment*	150	50	NS	VS	23		15 mo
Mostafa et al 14	2008	Prospective case series		121	45.5	63 M:58 F	VS = 60, vestibular neurectomies = 28, meningioma = 9, arachnoid cyst = 4	NS	NS	NS
Teo and Eljamel 22	2010	Prospective case series	*Craniotomy*	75	53	30 M:45 F	VS = 37, MVD = 30, other = 8	NS	NS	NS
			*Craniectomy*	30	66.3	13 M:17 F	VS = 20, MVD = 10	NS	NS	NS
Chovanec et al 13	2013	Prospective case series	*Endoscope assisted*	39	47	21 M:18 F	VS	26		26 mo
			*Standard*	50	45	30 M:20 F	VS	28		28 mo
Setty et al 12	2015	Prospective case series		12	46.7	8 M:4 F	VS	15	NS	NS
Yamakami et al 36	2004	Case series		89	51.5	42 M:47 F	VS	n-50 >3 cm n-39 <3 cm		
Shimanskiy et al 42	2016	Case series		176	47 b	50 M:126 F	VS	58% KOOS 4, 34.6% KOOS 3, 6.8% KOOS 2, 0.56% KOOS 1	NS	NS
Boghani et al 38	2013	Case series		7	51.9	1 M:1.33 F a	VS	NS	NS	22.9 mo

Abbreviations: BW, bone wax; CNV,—-; CSF, cerebrospinal fluid; F, female; LD, lumbar drain; M, male; MVD,—–-; NS, not specified; RS, retrosigmoid; VS, vestibular schwannoma.

a Value was averaged from a greater cohort.

b Median value.

### Repair Techniques

**Fig. 2:**
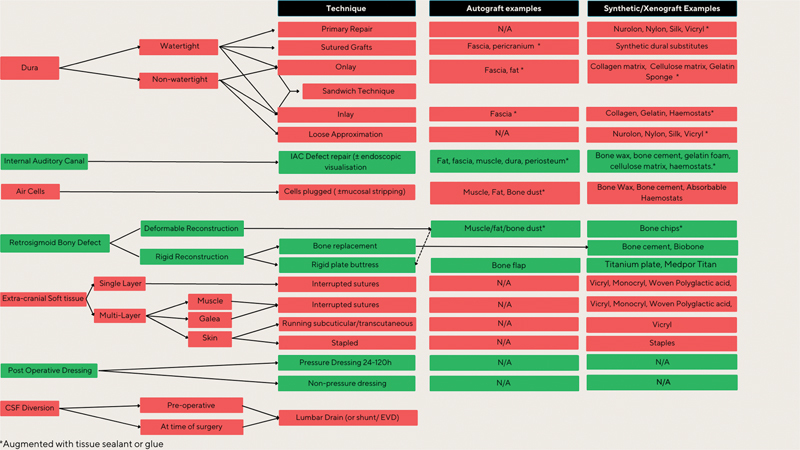
Taxonomy of available strategies used to prevent cerebrospinal fluid leaks during retrosigmoid vestibular schwannoma surgery. EVD, external ventricular drain; IAC, internal auditory canal; N/A, not applicable.

Each group reported unique approaches to repairing the skull base, with no two author groups reporting identical techniques and materials across stages of repair. Supplementary Table S3 (available in the online version only) synthesizes the materials and techniques used in all studies. Most preventative strategies focused on restoring the barriers to CSF flow, with few focusing on pressure-reducing strategies (i.e., CSF diversion via lumbar drains or external ventricular drains). Preventative strategies were seldom adapted to patient or intraoperative factors. Exceptions to this included the remnant subdural space, which was used to guide the placement of inlay grafts by Wong and Wong. 25 Overall, repair strategies were taxonomized into seven anatomical stages of repair. Fig. 2 provides a taxonomy of the repair strategies.

### Dura

The first possible distinction between the techniques used to repair the dura concerns whether they intend to achieve a watertight seal or not. Most studies 12 15 16 21 24 26 27 28 30 34 35 36 37 38 39 specified that their technique intended a watertight dural closure. Two studies 5 25 specified that their treatment of the dura was non-watertight. The remaining 25 studies either did not specify if the techniques was designed to be watertight or did not detail the dural treatment stage at all. Watertight approaches involved the combination of several strategies including primary sutured repairs, non-sutured onlays, non-sutured inlays, and sutured grafts. Non-watertight approaches involved the combination of techniques, including loose sutured approximation of the dural edges, with or without added inlays and onlays.

Primary repair was conducted without additional graft materials in eight studies. 5 16 19 22 28 32 39 40 Sutures were placed in an interrupted 21 31 32 41 or running 30 fashion, and materials included silk (3–0 19 or 4–0 5 ), nylon (4–0), 24 Nurolon (4–0), 24 Vicryl (4–0, 5–0), 21 26 41 and unspecified 6–0 sutures. 30 As outlined by Wong and Wong, watertight repair using sutures alone is difficult due to the thermal damage posed on the dural edges from bipolar cautery and the operating microscope, as well as physical damage from intentional dissection. 25 Therefore, if a watertight approach is intended, gaps in dural suture lines can be plugged with muscle 13 21 34 41 or fat, 34 and/or augmented using fibrin glue or tissue sealant (e.g., Tisseel 5 or Duraseal 32 ). Leonetti et al 28 performed an intraoperative Valsalva to check the dural seal prior to progressing beyond a primary sutured repair. Alternatively, primary repair techniques were commonly combined with other strategies such as inlays, onlays, or sutured grafts.

Non-sutured onlays were composed of either autologous or synthetic materials. Synthetic materials were absorbable dural substitutes composed of collagen of equine origin (e.g., Tachosil, 33 Tachocomb 42 ), collagen of bovine origin (e.g., Duragen 25 27 /Durepair 27 ), oxidized cellulose (e.g., Surgicel 31 ), gelatin sponge (e.g., Gelfoam 43 or Spongostan 5 ), or other non-specified synthetic dural substitutes. 21 24 Autologous onlays consisted of either fascia 35 or fat. 38 Onlay grafts were the only adjunct to primary dural repair in four studies. 12 20 35 43 Fibrin glue (e.g., Tisseel) was used to limit the mobility of onlay grafts in two studies. 15 21 Studies varied by the number of layers of onlay grafts used; for example, Goodarzi et al augmented a collagen onlay with an additional layer of Surgicel. 31 Overall, non-watertight onlays were more seldomly performed compared with the watertight alternatives. 5

Non-sutured subdural grafts (inlays) were described in four studies and consisted of both autologous and synthetic materials. 14 20 25 42 Autologous grafts (fascia lata) were used in one study by Mostafa et al, with no further augmentation techniques specified. 14 Synthetic materials included collagen matrix (e.g., Tissue Fleece 20 ), compressed gelatin sponge (e.g., Spongostan 20 ), non-compressed gelatin sponge (e.g., Gelfoam 25 ), or absorbable hemostats (e.g., Tachocomb 42 ). Three authors combined single or bi-layered inlays (composed of Tissue Fleece, 20 Spongostan, 20 Gelfoam, 25 or Tachocomb 42 ) with hemostatic onlays (Tachocomb 42 or Tachosil 20 ) or a collagen matrix graft (Duragen 25 ) with an intermediate primary dural repair, termed the “sandwich technique.” Arlt et al 20 compared the sandwich technique to a primary repair with a Tachosil onlay, yet did not find statistically significant differences in leak rates. Wong and Wong 25 performed the inlay component of the sandwich technique if there was a large subdural space.

To reduce the chance of onlay or inlay displacement, some authors sutured grafts into the dural edges. Materials used for sutured grafts included autografts (e.g., fascia 30 41 or pericranium 33 ), xenografts, 29 and synthetic dural substitutes. 7 Suture materials consisted of 4–0 Nurolon, 29 3–0 silk, 7 33 and 4–0 nylon. 7 Mastronardi et al harvested a 3 × 3 cm pericranial flap that was larger than the dural defect and inserted it as an “hourglass-shaped” plug, secured using running 3–0 silk and augmented by hemostatic onlays (Fibrillary Surgical and Tachosil). 33

### Internal Auditory Canal

The internal auditory canal (IAC) must frequently be drilled to achieve satisfactory intra-meatal resection during VS surgery. 44 However, this may expose air cell tracts through which CSF can egress to the middle ear. Techniques to repair the IAC employ both autologous and synthetic materials.

Synthetic materials used to repair the IAC included bone wax, bone cement, Gelatin foam (e.g., Gelfoam), Gelatin film (e.g., Gelfilm), oxidized cellulose (e.g., Surgicel), or absorbable hemostats (e.g., Tabotamp). Bone wax was the most commonly applied material and was used in isolation in three studies 18 26 45 yet was more commonly combined with both synthetic and autologous materials. Supplementary Table S3 (available in the online version only) details the many synthetic and autologous materials combined with bone wax to repair the IAC. Bone cement, composed of calcium phosphate (e.g., Cranios or Norian), was used to treat the IAC in three studies. 17 18 46 To address the potential drawback of bone cement causing nerve irritation, authors described reconstitution of the canal lumen using Gelfoam 18 46 or cotton balls 46 (which are later removed), protecting the nerves during layering. Baird et al 17 compared cement-based IAC treatment with their historical IAC repair protocol (bone wax, fat, muscle, and fibrin glue) and found a statistically significant reduction in CSF rhinorrhea ( *p* = 0.002) in the cement cohort. Hwa et al similarly compared the Cranios bone cement to bone wax and Norian bone cement and found statistically significant reductions in CSF leak rates ( *p* < 0.005). 18

Autologous grafts used to plug the IAC included muscle, 13 17 35 39 40 41 fat, 16 24 35 37 40 47 dura, 36 48 and periosteum. 37 To reduce the chance of dislodgement, muscle grafts were tacked with fibrin glue, 13 40 43 tissue glue, 49 or oxidized cellulose (e.g., Tabotamp 41 ). Daming et al premixed muscle, tissue glue, and Gelfoam into a paste, which was applied to the IAC defect. 39 Fat was used interchangeably with muscle in several studies 17 36 40 50 and thus was often secured with similar approaches. However, fat grafts could also be sewn into a preserved periosteal “saloon door” flap, as described by Fishman et al. 37 Yamakami et al used harvested dura to interpose IAC nerves and fat or muscle grafts to regain space in the IAC while sealing mastoid air cells. 36

Finally, endoscopes were used in four studies to aid the identification of potential air cell tracts. 13 40 49 51 Chovanec et al compared the CSF leak rate in endoscopic assisted and non-assisted procedures and suggested the endoscope may reduce the CSF leak rate as 5/39 of their endoscopic cohort had microscopically invisible air cells identified when the endoscope was used. 13 Drawbacks of the endoscope include the potential for neurovascular damage and heat injury. 13

### Air Cells

Twenty-eight studies reported their treatment of mastoid air cells, which included both autologous and synthetic materials. Some authors would strip the air cell mucosa prior to packing, citing a reduced risk of infection. 40 Synthetic materials consisted of bone wax (22/28), bone cement (4/28), and absorbable hemostats (1/28). Bone wax was used in isolation in 14/22 studies. 13 16 18 24 25 26 30 31 35 36 38 39 50 Bone wax was combined with autologous materials, including muscle (5/22), fat (2/22), and bone dust (1/22), and synthetic materials such as bone cement (3/22). Bone cement was composed of calcium phosphate in three of three studies specifying cement composition.

Autologous materials used to fill air cells included muscle (7/28), fat (5/28), and bone dust (1/28). To reduce the potential drawback of dislodgement, several studies adjuncted these materials with fibrin glue. 17 20 40 43 45 49 51 Bani et al 41 used muscle and an absorbable hemostat (Tabotamp). Lüdemann et al 23 compared fat and muscle based air cell packing (tacked with fibrin glue) in a retrospective case series and found that fat was associated with reduced CSF leak rates, albeit without statistical significance ( *p* = 0.09).

### Retrosigmoid Skull Defect

Multiple techniques used to restore the bony RS defect were described. The first branching point between the techniques considers whether the reconstructions were performed using materials that were deformable or non-deformable.

Three studies described a deformable reconstruction technique. 18 22 28 This approach does not attempt to restore a hard skull substitute but instead repairs the bony defect with fat, 28 muscle, 18 or bone dust (mixed with tissue glue) 49 followed by soft tissue closure. The reported drawback of deformable reconstructions is that the temporal soft tissue may form adhesions to the dura, increasing postoperative headaches. 22 Indeed, Teo and Eljamel 22 compared a deformable closure to an autologous bone flap closure and found statistically significant reductions in postoperative headaches with non-deformable closures.

Non-deformable reconstructions describe a bony defect repair using hard, bony substitutes or buttress plates. Non-deformable bone substitutes consist of bone cement composed of either calcium phosphate (e.g., Norian, 18 Cranios, 18 46 or Hydroset 52 ) or polymethacrylate (e.g., Palacos 20 21 40 43 ). Bone cement was cited to offer benefits over alternative closure techniques due to excellent tensile strength, improved cosmesis, and reduced headaches. 53

An alternative non-deformable reconstruction technique is to use either synthetic or autologous bone plates, with or without repairing the underlying bony defect with soft tissue. Autologous bone flap replacement was described in 10 studies. 15 20 21 22 33 36 37 51 Techniques to secure the bone flap included either silk sutures 5 or plate and screws. 5 19 30 Teo and Eljamel secured the autologous bone flap through dural tent sutures. 22 Some studies 13 22 47 augmented autologous plate closures using bone dust to close the remnant gaps in the skull. Synthetic alternatives to an autologous bone flap included titanium mesh plates, with or without porous polyethylene coating (Medpor titan), and an artificial bone flap (Biobone 36 ). Titanium was the most common material, used in six of nine studies reporting the use of synthetic plates. 25 29 31 32 33 39 Medpor titan was used in three studies 24 34 38 and is cited for having improved biocompatibility compared with pure titanium plates, promoting growth into the plate. Synthetic plates were either used in isolation, covered with bone cement, 29 or used as a buttress for a medial graft, serving to prevent architectural disruption. The latter approach involved the use of fat, 24 38 bone chips, 31 or Gelatin foam. 25 32 The purpose of these materials is to improve the seal in medial anatomical regions (dura, IAC, air cells), and the plate provides a supportive buttress.

### Extracranial Soft Tissue

The extracranial soft tissue may be closed in a single layer or in multiple layers. Of the studies specifying their closure technique, a multilayered closure was most common. The separate layers described in multilayered repairs constituted the muscle, galea, and skin. Muscle layers were closed in an interrupted fashion with absorbable sutures, composed of 2–0 Vicryl, 29 3–0 Vicryl, 31 3–0 Monocryl, 15 or 2–0 woven polyglactic acid. 48 The galeal layer was closed using 2–0 Vicryl 29 or 3–0 Vicryl 31 and only an interrupted technique was specified. The approach to the skin was a running subcuticular, 25 30 a running transdermal, 32 or stapled closure. 29 30 Skin suture materials included 4–0 nylon, 29 4–0 Monocryl. 15 31 Skin glue (Dermabond) was used to augment sutured closure in one study. 25 Overall, detailed descriptions of the extracranial soft tissue closure techniques were infrequently provided. Instead many studies reported a “multilayered closure”; thus, it is likely that the nuances of certain closures were not captured.

### Postoperative Dressings and Positioning

Nine studies specified the application of dressings to the wound, which consisted of either pressure or non-pressure dressings. The former was described in four studies, 21 26 28 34 with a length of application ranging from 48 hours 21 to 120 hours. 28 The purported benefit of pressure dressings is to prevent CSF leaks or subdural collections by restoring the pressure gradient established across the cranial interface, otherwise thought to propagate CSF-related complications. 54 Two studies 25 48 specified that they applied nonpressure dressings for an unspecified length of time, while the remaining studies did not specify dressing usage at all. Venable et al placed a Dermabond tissue adhesive as their only dressing. 32

### CSF Diversion

Twelve out of 42 studies reported whether they employed a CSF diversion strategy to reduce the incidence of CSF leaks. Seven out of 12 studies specified that they did not routinely perform perioperative CSF diversion unless a postoperative leak was identified. At the preoperative stage, some patients may have a Lumbar Drain or Ventriculo-Peritoneal shunt placed to reduce the CSF pressure, although this is uncommon. Brennan et al 35 inserted a lumbar drain at the time of induction and removed this at the end of surgery. Mastronardi et al placed a lumbar drain for larger tumors (>2.5 cm) for 3 to 4 days, draining 10 mL/hour. 33 Magill et al 29 placed an intraoperative lumbar drain or EVD in 5% of cases, without specifying the indications for such an approach. Leonetti et al 28 performed lumbar drainage in all RS cases for 24 to 48 hours. Crowson et al performed a retrospective case–control comparing RS VS surgery with and without lumbar drainage and found that preoperative lumbar drainage did not influence CSF leak rates in their cohort. 9

### CSF Leak Rates

A CSF leak was defined as rhinorrhea, otorrhea, or incisional leaks. The median overall CSF leak rate with unique repair protocols was 6.3% (IQR: 1.3–8.4%). The incidence rates of rhinorrhea, incisional leaks, and otorrhea were 1.5% (IQR: 0–5%), 0% (IQR: (0–3.6%), and 0% (IQR: 0–0%), respectively. Four studies reported leak rates of 20% or above in at least one of their cohorts. Jung et al, reported eight cases of rhinorrhea out of 30 operations (26.5%); however, they notably defined CSF leaks as overt leaks of pseudomeningoceles (unlike other studies which provided separate values) which may account for the increased number. 47 Teo and Eljamel reported six leaks in 30 patients (20%) and hypothesized possible factors accounting for the higher leak rate being the lack of bone flap as well as a larger sized cranial opening. 22 Similarly Della Pepa et al found five leaks in a cohort of 20 patients and also reported this to be due to the lack of a replaced bone flap. 19 Chovanec reported 10 leaks in 50 patients and attributed this to the lack of endoscopic visualization in this cohort. 13 CSF leaks were diagnosed by gross visualization of CSF egress, with or without a Valsalva maneuver 26 40 and through biochemical confirmation tests (e.g., β-2 transferrin, 26 or glucose 40 ). The standard treatment protocols for confirmed CSF leaks varied between studies and were scarcely reported. Some authors 26 41 opted for initial “conservative management,” yet this had varied definitions, including various combinations of bed rest, head elevation, compression dressings, wound suturing, 15 21 and lumbar drains. Therapeutic lumbar drains were used in 19 of 39 studies. 5 7 17 18 19 20 21 25 26 35 36 37 39 40 41 43 47 48 Surgical repair was required for at least one CSF leak in 16 of 39 studies.

## Discussion

### Principal Findings

We performed a systematic review of 42 studies to outline the breadth of repair strategies deployed for the lateral skull base following RS VS surgery. The motivation for this review was to support the ongoing efforts to mitigate CSF leaks following RS VS surgery, a complication that carries profound implications. The potential issues associated with CSF leaks are multifold, ranging from low-pressure headaches and pneumocephalus to potentially life-threatening meningitis. 55 Additionally, CSF leaks may require revision surgeries, thereby escalating healthcare expenditures and prolonging length of stay. An estimation by Chern et al placed the median cost of a CSF leak repair at $50,401 (notably in an American healthcare setting). 6 Although various risk factors have been recognized in association with CSF leaks, the repair protocol employed intraoperatively is particularly important. This review classified the intraoperative repair protocols into seven stages, namely the dura, IAC, air cells, RS bony defect, extracranial soft tissue, postoperative dressings, and CSF diversion strategy. Key findings are outlined below.

Dural strategies were specified in 36 studies and resulted in 29 combinations of autografts, xenografts, and synthetic substitutes used in primary repairs, onlays, inlays, and sutured grafts. IAC treatment was described in 19 studies, resulting in 19 permutations of autologous grafts (e.g., muscle, fat) and synthetic materials (e.g., bone wax, bone cement, glue, gelatin film, and hemostats), which were used to reconstruct the IAC canal and occlude potential air cells tracts, with or without endoscopic visualization. Mastoid air cell treatment was described in 31 studies, producing 9 combinations of packing materials. The most frequent material was bone wax (23/31 studies), followed by muscle (4/31 studies), fat (4/31 studies), bone cement (4/31 studies), or bone paste (1/31 studies). The RS skull defect repair was specified in 32 studies and resulted in 15 different combinations of either deformable or non-deformable reconstruction techniques consisting of combinations of soft tissue, bone cement substitutes, or rigid plate buttresses. The extracranial soft tissue wound was predominantly closed in layers, varying mainly by the suture materials used. CSF diversion techniques were infrequently specified as a strategy but could consist of preoperative or intraoperative use of lumbar drains, shunts, or EVDs. Postoperative dressings and head positioning were seldomly reported.

Overall, there was considerable inter- and intra-institutional heterogeneity. Regarding the former, no two groups implemented identical techniques and materials across all stages. However, this disparity was more pronounced in some stages than others. Stages with the least heterogeneity were the closure of extracranial soft tissue, typically achieved in two or three layers, and CSF diversion, which was neither frequently reported nor performed and ultimately binary in nature (performed or not performed). Conversely, stages with a high degree of heterogeneity included the approaches to the dura and IAC, as demonstrated by their proportion of technique permutations relative to the overall study number being 75% (27 combinations in 36 studies) and 100% (19 combinations in 19 studies), respectively. Stages with moderate heterogeneity included the mastoid air cells and RS skull defect, as 9 and 15 combinations were generated from 32 and 31 studies, respectively. Additionally, there was intra-institutional variation. For instance, among the 36 studies describing the treatment of the dura, five suggested variations in practice at their institution, with surgeon preference serving as the determinant. 25 28 33 34 52 Such discrepancies underscore the uncertainty surrounding the optimal repair strategy following RS VS surgery.

Additionally, this review identified inconsistent reporting across repair stages. Specifically, only 2 out of 42 studies 24 51 detailed a protocol considering all seven stages of the repair. Excluding studies that only omitted their CSF diversion protocol improves this total of comprehensive reports to seven. Such discrepancy in reporting precludes a meaningful comparative analysis of repair strategies, as one cannot account for the effect of unreported closure strategies. While it may be argued that the lack of reporting of a repair stage should warrant the assumption that this stage was not repaired at all, the number of studies in our analysis not reporting wound closure demonstrates that the lack of reporting does not equate to the lack of treatment.

The observed heterogeneity reflects the fact that there is a dearth of high-level evidence directing the best approach to prevent CSF leaks post–RS VS surgery, calling for a novel study design. Indeed, the retrospective nature of most of our included studies and the inconsistent reporting of both repair strategies and key data points makes it difficult to perform a meaningful comparative analysis. Instead, we recommend that prospective, multicenter, observational service evaluations be established with the intention of capturing a broad scope of potential risk factors for CSF leaks in RS VS surgery, including all stages of the intraoperative repair protocols. Such designs have successfully been used to identify repair protocols associated with leak-free endonasal skull base surgery—namely, gasket seals and lumbar drains. 56 Through such evaluations, one may successfully account for the true complexity and interactions of the many factors that predict whether a postoperative CSF leak is experienced following RS VS surgery.

### Comparison to Current Literature

To our knowledge, this is the first systematic review that describes the breadth of operative repair protocols employed internationally following RS VS surgery. Previous work by Layard Horsfall et al established a UK-based, consensus-derived, codified operative workflow for RS VS surgery, which helpfully delineated not only the closure phase but all 40 steps of the operation. 44 This work expands on that by Layard Horsfall et al, by broadening the scope of the closure phase from a UK-centric to an international perspective. Additionally, we focused solely on the closure phase and thus were able to delineate subtypes of repair techniques in each anatomical stage. Additionally, we report on the use of materials not described in previous work, such as fat grafts, xenografts, collagen grafts, and subtypes of bone cement. Other narrative reviews summarizing prophylactic strategies for CSF leaks in RS VS surgery include the one by Safdarian et al, which compared the sitting versus lateral positioning of patients during VS surgery and found no difference in leak rates. 57

### Strengths and Limitations

This systematic review benefits from its systematic design. Through a pragmatic inclusion of studies that specified their relevance to VS surgery, without precluding those with non-VS indications, a comprehensive capture of the breadth of surgical practice in RS VS surgery was possible. Our taxonomy categorizes the heterogenous repair protocols into seven stages, and aims to highlight important variations. However, the true influence of individual repair techniques may span across multiple stages, which is not reflected in the taxonomy. For instance, the materials used to repair the bony RS defect (e.g., HAC) will inevitably influence the seal achieved at other stages, such as the dura or air cells. Additionally, the designs of the included studies were predominantly observational and retrospective, leading to the risk of selection bias, information bias, measurement error, and confounders. 58 Furthermore, studies reporting surgical outcomes are prone to publication bias. 59 Finally, there was inconsistent reporting of key outcome measures, including patient demographics (sex, age, body mass index), tumor size, repair protocols, and CSF leak diagnostic modalities. Ultimately, this prevented a comparative meta-analysis of repair protocols.

## Conclusion

The intraoperative strategies used to prevent CSF leaks during RS VS surgery vary significantly between institutions. The present systematic review classified the heterogenous repair protocols into an intuitive taxonomy with seven stages of repair described. However, comparative analyses were not possible due to heterogeneity in reporting of key outcomes. Future prospective observational evaluations are required to accurately capture a comprehensive selection of potential CSF risk factors, including all stages of the operative repair protocols.
