# Supplementary material for: Skull Base Repair following Resection of Vestibular Schwannoma: A Systematic Review (Part 1: The Retrosigmoid Approach)
Source: J Neurol Surg B Skull Base. 2024 Jan 22;85(Suppl 2):e117–30. doi: 10.1055/a-2222-0184 (PMC11495915; doi:10.1055/a-2222-0184)
Supplement: Supplementary file 1 — Supplementary Material [file 10-1055-a-2222-0184-s23sep0149.pdf]

Supplementary Table S1 Ovid Medline All + Embase

Term	Keywords
1	(Retrosigmoid or translabyrinthine or “vestibular schwannoma” or “lateral skull base” *or* “acoustic neuroma”).mp. [mp = ti, ab, hw, tn, ot, dm, mf, dv, kf, fx, dq, bt, nm, ox, px, rx, ui, sy, ux, mx]
2	(“Cerebrospinal fluid” or CSF or leak or pyorrhoea or rhinorrhoea or “wound leak” or otorrhoea).mp. [mp = ti, ab, hw, tn, ot, dm, mf, dv, kf, fx, dq, bt, nm, ox, px, rx, an, ui, sy, ux, mx]
3	1 and 2

Supplementary Table S2 Repair techniques classified according to stages of repair, ordered sequentially by CSF leak (%)

Authors	Patients (*n*)	CSF leak (%)	CSF leak (*n*)	Dura	Internal acoustic canal	Air cells	RS skull defect	Soft tissue	CSF diversion
Yang et al 2023	16	0.0%	0	Primary closure	Endoscopic inspection. Bone wax + muscle + biological protein glue	Bone wax + muscle fragments + biological protein glue	Autologous bone replaced	Multilayered closure	NS
Venable et al 2018	86	0.0%	0	Primary repair (interrupted 4–0 Nurolon) + DuraSeal tissue glue	NS	Bone wax *or* bone paste	Gelfoam + titanium plate	Multilayered closure	Not performed
Setty et al 2015	12	0.0%	0	Onlay graft—no further specification	NS	NS	Bone cement	Multilayered closure (absorbable sutures)	NS
Mastronardi et al 2016	27	0.0%	0	Sutured inlay hourglass-shaped plug (pericranium graft + 3–0 running silk) + absorbable hemostats (fibrillary Surgicel) + small pieces of hemostat (TachoSil) + dural sealant (DuraSeal)	NS	NS	Autologous bone replaced *or* titanium plate	NS	LD for larger tumors (>2.5 cm) and left in place for 3–4 days
Luryi et al 2017	20	0.0%	0	Primary repair *or* overlay (processed collagen implant: DuraGen/ Durepair)	NS	Bone cement: calcium phosphate (HydroSet)	Bone cement (HydroSet)	Multilayered closure. Periosteum + muscle + deep dermal + skin	Not performed
Ling et al 2014	58	0.0%	0	Primary repair (4–0 Nurolon) +- tissue dural sealants	Bone wax + fat	Bone wax	Fat graft + Medpor Titan plate	Multilayered closure	Not performed
Goodarzi et al 2018	25	0.0%	0	Primary repair (interrupted, 4–0 braided nylon) + overlay (collagen + Surgicel)	NS	Bone wax	Autologous bone chips/ dust + cellulose matrix + titanium plate	Multilayered closure. Muscle (3–0 Vicryl) + deep fascia (3–0 Vicryl) + dermis (inverted 4–0 Vicryl) + skin (4–0 nylon)	NS
Della Peppa et al 2011	14	0.0%	0	Primary repair (3–0 silk) + fibrin glue	NS	NS	Autologous bone flap replaced	NS	NS
Cueva et al 2005	115	0.0%	0	Primary repair (4–0 woven nylon) + onlay (blood-impregnated	Bone wax + dura	Bone wax	NS	Multilayered closure. Muscle (interrupted 2–0 woven	NS

(*Continued*)

 © 2024. The Author(s).

Supplementary Table S2 (*Continued*)

Authors	Patients (*n*)	CSF leak (%)	CSF leak (*n*)	Dura	Internal acoustic canal	Air cells	RS skull defect	Soft tissue	CSF diversion
				microfibrillar collagen hemostat)				polyglactic acid suture) + deep dermal layer (inverted, interrupted 2–0 woven polyglactic acid suture) + skin (staples)	
Boghani et al 2013	7	0.0%	0	Watertight dural closure + fat	Bone wax + fat	Bone wax + fat	Fat + Medpor Titan plate	Multilayered closure	NS
Azad et al 2016	24	0.0%	0	Primary closure (4–0 Nurolon) + reinforcement with muscle or fat for small additional defects	Bone wax + fat + Surgicel + fibrin glue	Bone wax	Fat + Medpor Titan plate	Multilayered closure	Not performed
Mostafa et al 2008	121	0.0%	0	Inlay (fascia lata)	NS	NS	NS	Two-layered closure	NS
Wong et al 2023	114	0.9%	1	Sandwich technique: If a large subdural space: inlay (non-compressed absorbable gelatin sponge: Gelfoam) + primary approximation (interrupted Nurolon + onlay (collagen matrix graft: DuraGen)	NS	Bone wax	Gelatin foam + titanium plate	Multilayered closure. Suboccipital muscle (absorbable sutures) + Galea-fascial plane + skin (running sub-cuticular suture and skin glue—Dermabond)	NS
Songyu et al 2019	97	1.0%	1	Primary repair (running sutures) + sutured patch (fascia + 6–0) + overlay (absorbable dural substitutes)	NS	Bone wax	Autologous bone flap secured with screws	Multilayered closure. Muscles and fascia: interrupted 2–0 absorbable sutures. S/c tissue and galea: 3–0 interrupted absorbable sutures. Skin: staples	NS
Hwa et al 2021	101	1.0%	1	The dura over the posterior fossa was then closed in a water-tight manner	Bone wax + Gelfilm and Gelfoam to recreate canal + bone cement (Cranios)	NS	Bone cement: calcium phosphate (Cranios)	Multilayered closure	NS
Shimanskyi et al 2016	176	1.7%	3	Sandwich technique: Inlay (Tachocomp sponge patch) + primary dural closure with 7 mm gaps (absorbable and nonabsorbable sutures) + overlay (tachocomb sponge)	NS	NS	NS	NS	NS

 © 2024. The Author(s).

Supplementary Table S2 (*Continued*)

Authors	Patients (*n*)	CSF leak (%)	CSF leak (*n*)	Dura	Internal acoustic canal	Air cells	RS skull defect	Soft tissue	CSF diversion
Samii et al 2006	200	2.0%	4	Gelfoam onlay	Muscle + fibrin glue	Muscle + fibrin glue + (rarely bone wax)	Bone cement: methyl methacrylate (Refobacin-Palacos)	NS	NS
Lüdemann et al 2008	137	2.2%	3	NS	NS	Fat + fibrin glue	NS	NS	NS
Changjiang et al 2020	*136*	2.2%	3	Primary repair (interrupted 4–0 coated Vicryl) + muscle pieces used to seal the dural defects (if present) + onlay nonsewable dural substitute (TianXinFu) + fibrin glue (Shanghai RAAS)	NS	NS	PMMA cement (Palacos)	Multilayered closure: muscle + fascia + skin	NS
Yamakami et al 2004	89	2.3%	2	Water tight closure	Dura + muscle or fat	NS	autologous bone flap OR artificial bone flap (Biobone)	NS	NS
Chibbaro et al 2019	40	2.5%	1	Primary closure + "patch" overlay + fibrin glue	NS	NS	Autologous bone flap replaced	C-shaped incision. Multilayered closure. Muscle (3–0 Monocryl) + S/c tissue (3–0 Monocryl) + skin (4–0 Monocryl *or* staples)	NS
Zhang et al 2021	177	3.4%	6	NS	Gelfoam or cotton ball placed in IAC to recreate canal + bone cement: calcium phosphate (Cranios) applied + once cement dry, Gelfoam or cotton ball removed	Bone cement: calcium phosphate (Cranios)	Filled with cement: calcium phosphate (Cranios)	Multilayered closure	NS
Stieglitz et al 2011	519	4.2%	22	Primary repair + fibrin-glue	Endoscope visualization. Fat *or* muscle + fibrin-glue	Mucosa stripped. Fat tissue + fibrin glue	Bone cement: polymethylmethacrylate bone cement (Palacos)	Multilayered closure	NS
Magill et al 2022	40	5.0%	2	Sutured patch (interrupted 4–0 Nurolon, xenograft) + dural sealant (Adherus)	NS	Bone wax	Titanium plate + bone cement (HydroSet)	Multilayered closure. Fascia: interrupted 2–0 Vicryl . S/C tissue and galea: running 2–0 Vicryl. Skin: 4–0 nylon	4 cases (10%). Lumbar SA drain = 2, EVD = 2
Cui et al 2014	37	5.4%	2	Primary dural repair	Mixed muscle and glue and Gelfoam	Bone wax	Titanium plate	NS	NS
Lüdemann et al 2009	283	5.7%	16	NS	NS	Muscle + fibrin glue	NS	NS	NS

(*Continued*)

 © 2024. The Author(s).

Supplementary Table S2 (*Continued*)

Authors	Patients (*n*)	CSF leak (%)	CSF leak (*n*)	Dura	Internal acoustic canal	Air cells	RS skull defect	Soft tissue	CSF diversion
Schackert et al 2021	544	6.1%	33	NS	Endoscopic inspection. Muscle + tissue glue	Bone wax + muscle + fibrin glue	Bone dust + tissue glue *or* autologous bone flap replaced	NS	NS
Bani et al 2002	224	6.3%	14	Primary repair (4–0 interrupted Vicryl) + gaps filled with muscle + collagen sponge overlay	Muscle + Tabotamp	Muscle + Tabotamp	Bone dust	Multilayered closure (absorbable sutures)	NS
Teo et al 2010	75	6.7%	5	Primary repair	NS	NS	Autologous bone flap replaced, sutured to the dura with a tent suture + bone dust	NS	NS
Arlt et al 2013	41	7.3%	3	Sandwich technique: Inlay (equine collagen: Tissue fleece and gelatin sponge: Spongostan) + primary repair + onlay (Tachosil)	NS	muscle (neck) + fibrin glue	Autologous bone flap replaced *or* Bone cement: polymethacrylate (Palacos)	NS	NS
Chibbaro et al 2018	40	7.5%	3	Primary repair + "patch" overlay + fibrin glue	NS	NS	Autologous bone flap replaced	Lazy S incision. Multilayered closure. Muscle (3–0 Monocryl) + S/c tissue (3–0 Monocryl) + skin (4–0 Monocryl Tabotamp staples)	NS
Chovanec et al 2012	39	7.7%	3	Primary repair (absorbable suture) + muscle/fascia-technique not clear + fibrin glue	Endoscopic inspection. Muscle + fibrin glue	Bone wax	Autologous bone flap + bone pate	NS	Not performed
Leonetti et al 2001	191	7.8%	15	Primary repair *or* fascial patch (unclear technique)+ intraoperative Valsalva to check completeness of intraoperative repair	Bone wax + bone pate + temporalis muscle + dural flap approximation (6–0 nylon)	NS	Fat + soft tissue closure	NS	Performed intraoperatively, continued 24–48 h
Bayazit et al 2009	412	7.8%	32	Primary repair (5–0 sutures)	Bone wax	Bone wax	NS	Multilayered closure	NS
Hwa et al 2021	63	7.9%	5	The dura over the posterior fossa was then closed in a water-tight manner	Bone wax + Gelfilm and Gelfoam to recreate canal + bone cement (Norian)	Bone wax + bone cement (Cranios/Norian)	Filled with cement: hydroxyapatite (Norian)	Multilayered closure	NS
Brennan et al 2000	151	8.0%	12	Primary repair (watertight) +- onlay (fascia)	NS	Bone wax	NS	NS	Intraoperative LD

 © 2024. The Author(s).

Supplementary Table S2 (*Continued*)

Authors	Patients (*n*)	CSF leak (%)	CSF leak (*n*)	Dura	Internal acoustic canal	Air cells	RS skull defect	Soft tissue	CSF diversion
Chang-jiang et al 2019	*107*	8.4%	9	Primary repair (interrupted 4–0 coated Vicryl) + muscle pieces used to seal the dural defects (if present) + onlay non-sewable dural substitute (TianXinFu) + fibrin glue (Shanghai RAAS)	NS	NS	Autologous bone flap replaced	Multilayered closure: muscle + fascia + skin	NS
Kalamarides et al 2004	59	8.5%	5	Primary repair (watertight)	Fat	Bone wax	NS	NS	NS
Fishman et al 2004	71	8.5%	6	Watertight closure with temporalis graft—no further specification.	Fat+ "saloon door" flap of periosteum + fibrin glue	Bone wax + fat	Autologous bone flap	NS	NS
Satha-porn-theera et al 2020	286	9.1%	26	**Left open:** primary dural approximation + Gelfoam sponge (SPONGOSTAN) Primary repair: watertight primary dural closure (silk 4–0). Primary repair with glue: watertight primary dural closure (silk 4–0) + fibrin glue sealant (TISSEEL)	NS	NS	Craniotomy: autologous bone flap with or without fixation (fixation with silk, wire, or screws) *or* Craniectomy: left skull defect opens after dural closure	NS	NS
Crowson et al 2015	130	9.2%	12	NS	NS	NS	NS	NS	With and without LD
Montano et al 2021	103	9.7%	10	Sutured patch (SDS) (3–0 silk or 4–0 nylon)	NS	NS	NS	NS	NS
Becker et al 2003	100	10.0%	10	NS	Endoscopic visualization in some cases. BW + Muscle *or* fat	Bone wax	NS	NS	Not performed
Baird et al 2007	130	10.0%	13	NS	Bone cement	Bone wax + bone cement	NS	NS	Not performed
Arlt et al 2013	40	10.0%	4	Primary repair + onlay (Tachosil)	NS	Muscle (neck) + fibrin glue	Autologous bone flap replaced *or* bone cement: polymethacrylate (Palacos)	NS	NS
Plain-fossé et al 2021	175	11.4%	20	SDS patch—unclear if sutured	Bone wax	bone wax + bone dust *or* occasionally with muscle and fibrin glue	NS	Multilayered closure	NS
Baird et al 2008	150	18.7%	28	NS	Bone wax +- fat +- muscle +- fibrin glue	BW +- fat +- muscle +- fibrin glue	NS	NS	Not performed

(*Continued*)

Supplementary Table S2 (*Continued*)

Authors	Patients (*n*)	CSF leak (%)	CSF leak (*n*)	Dura	Internal acoustic canal	Air cells	RS skull defect	Soft tissue	CSF diversion
Hwa et al 2021	32	18.8%	6	The dura over the posterior fossa was then closed in a water-tight manner	Bone wax	Bone Wax	Filled with muscle+ Dura-Seal/Covidien	Multilayered closure	NS
Teo et al 2011	30	20.0%	6	Primary repair	NS	NS	Craniectomy	NS	NS
Chova-nec et al 2013	50	20.0%	10	NS	Muscle + fibrin glue	Bone wax	NS	NS	NS
Della Peppa et al 2011	20	25.0%	5	Primary repair (3–0 silk) + fibrin glue	NS	NS	Craniectomy	NS	NS
Jung et al 2000	30	26.7%	8	Pericranial graft used—technique not specified	Fat	NS	Autologous bone flap + bone dust	NS	NS

Abbreviations: BW, bone wax; CSF, cerebrospinal fluid; LD, lumbar drain; NS, not specified; RS, retrosigmoid.

Supplementary Table S3 Risk of bias and quality assessment

Bias domains and scoring:

A. Description of patient: details provided of patient characteristics.
   i. High risk of bias (scored 1): no description of patient demographics and CSF leak risk factors (i.e., patients underwent VS resection).
   ii. Low risk of bias (scored 0): clear description of patient demographics, including potential risk factors (i.e., BMI, tumor size, etc.).

B. Description of pathologies: details provided of lesions.
   i. High risk of bias (scored 1): no description of conditions for selection (i.e., VS, meningioma, and other).
   ii. Low risk of bias (scored 0): clear description of cohort (i.e., VS = 100, meningioma = 5)

C. Description of repair technique I: clarity of description and identifiability of each treatment group.
   i. High risk of bias (scored 1): heterogenous repair strategies with breakdown of case composition the repair technique were used (i.e., repair technique A or B or C were used based on surgeon preference).
   ii. Low risk of bias (scored 0): description in which percentage/proportion of cases a repair technique was used (e.g., repair A was used in for tumors >2.5 cm [n = 6] or repair B was used in all cases).

D. Description of repair technique II: clarity of description of technique and materials.
   i. High risk of bias (scored 1): minimal detail of repair technique described (e.g., dura was closed using fascia).
   ii. Low risk of bias (scored 0): clear description of all stages of skull-base repair (dura, IAC, air cells, skull, and soft tissue).

E. Separate reporting of postoperative pseudo-meningoceles, rhinorrhea, incisional leaks and otorrhea.
   i. High risk of bias (scored 1): no distinction of pseudomeningoceles and CSF leaks.
   ii. Low risk of bias (scored 0): report separately on CSF leaks (rhinorrhea, incisional leak, otorrhea) and pseudomeningoceles or provide a clear description of the definition of a CSF leak.

 © 2024. The Author(s).

Scoring per paper:

Author	Year	PMID	A	B	C	D	E	Total
Magill et al	2022	35433187	1	0	0	0	0	1
Yang et al	2023	36733306	0	0	0	0	0	0
Plainfossé et al	2021	33942123	0	0	0	1	1	2
Montano et al	2021	34221589	1	0	0	1	0	2
Schackert et al	2021	33383200	0	0	0	1	1	2
Zhang et al	2021	34121078	0	0	1	1	1	3
Sathaporntheera et al	2020	a	0	0	0	1	0	1
Songyu et al	2019	31033764	1	0	0	1	0	2
Hwa et al	2021	33481543	0	0	0	0	0	0
Boghani et al	2013	23371869	1	0	0	0	0	1
Della Pepa et al	2011	21805283	1	0	0	0	1	2
Stieglitz et al	2010	21099580	0	0	0	0	0	0
Goodarzi et al	2018	30210980	1	0	0	1	0	2
Venable et al	2018	**30009112**	1	0	0	0	0	1
Mastronardi et al	2016	27069742	1	0	0	1	0	2
Setty et al	2015	262255307	0	0	0	1	0	1
Ling et al	2014	25091535	1	0	0	0	0	1
Cui et al	2014	24514888	0	0	0	1	1	2
Arlt et al	2013	**22080996**	0	0	0	1	0	1
Stieglitz et al	2011	**21862208**	1	0	1	0	1	3
Wong et al	2023	**36870088**	0	0	0	1	1	2
Chovanec et al	2013	**23010789**	1	0	0	1	0	2
Bayazit et al	2009	**20068375**	1	0	0	1	1	3
Mostafa et al	2008	**19412406**	1	0	0	1	1	3
Chibbaro et al	2018	**29258939**	1	0	0	1	0	2
Luryi et al	2017	**28390811**	1	0	0	1	0	2
Azad et al	2016	**26482457**	1	0	1	0	0	2
Shimanskyi et al	2016	**27801403**	1	0	0	1	0	2
**Jung et al**	2000	**10825523**	1	0	1	1	0	3
Brennan et al	2001	**11213957**	1	0	1	1	0	3
Bani et al	2002	**12382125**	1	0	0	0	1	2
Becker et al	2003	**12544038**	1	0	1	1	1	4
Kalamarides et al	2004	**15354009**	1	0	0	1	1	3
Yamakami et al	2004	**14966164**	1	0	0	1	1	3
Fishman et al	2004	**15091225**	1	0	0	1	0	2
Cueva et al	2005	**16272937**	1	0	0	1	1	3
Samii et al	2006	**17044553**	1	0	0	1	1	3
Baird et al	2007	**17695389**	1	0	0	1	0	2
Changjiang et al	2019	**31137454**	1	0	0	1	1	3
Leonetti et al	2001	**11337653**	1	0	1	0	1	3
Teo et al	2010	**20871444**	1	0	0	1	1	3
Lüdemann et al	2008	**18728602**	1	0	0	1	1	3
Crowson et al	2015	**26239000**	0	0	1	1	0	2

[a]doi.org/10.1016/j.inat.2020.100865.

 © 2024. The Author(s).
